# Pregnancy and the Use of Disease-Modifying Therapies in Patients with Multiple Sclerosis: Benefits versus Risks

**DOI:** 10.1155/2016/1034912

**Published:** 2016-12-18

**Authors:** Raed Alroughani, Ayse Altintas, Mohammed Al Jumah, Mohammadali Sahraian, Issa Alsharoqi, Abdurahman AlTahan, Abdulkader Daif, Maurice Dahdaleh, Dirk Deleu, Oscar Fernandez, Nikolaos Grigoriadis, Jihad Inshasi, Rana Karabudak, Karim Taha, Natalia Totolyan, Bassem I. Yamout, Magd Zakaria, Saeed Bohlega

**Affiliations:** ^1^Division of Neurology, Amiri Hospital and Division of Neurology, Dasman Diabetes Institute, Dasman, Kuwait; ^2^Division of Neurology, Cerrahpasa School of Medicine, Istanbul University, Istanbul, Turkey; ^3^King Abdullah International Medical Research Center, King Saud Ben Abdulaziz University for Health Sciences, NGHA, Riyadh, Saudi Arabia; ^4^KFMC, Ministry of Health, Riyadh, Saudi Arabia; ^5^MS Research Center, Neuroscience Institute, Tehran University of Medical Sciences, Tehran, Iran; ^6^Clinical Neurosciences Department, Salmaniya Medical Complex, Manama, Bahrain; ^7^Neurology Section, King Khalid University Hospital, King Saud University and Dallah Hospital, Riyadh, Saudi Arabia; ^8^King Khalid University Hospital, King Saud University, Riyadh, Saudi Arabia; ^9^Department of Internal Medicine, Neurology Section, Arab Medical Center and Khalidi Hospital, Amman, Jordan; ^10^Department of Neurology (Medicine), Hamad Medical Corporation, Doha, Qatar; ^11^Instituto de Investigación Biomédica de Málaga (IBIMA), Hospital Regional Universitario, Málaga, Spain; ^12^Laboratory of Experimental Neurology and Neuroimmunology, Multiple Sclerosis Center, 2nd Department of Neurology, AHEPA University Hospital, Aristotle University of Thessaloniki, Thessaloniki, Greece; ^13^Neurology Department, Rashid Hospital and Dubai Medical College, Dubai Health Authority, Dubai, UAE; ^14^Department of Neurology, Neuroimmunology Unit, Hacettepe University Hospitals, Ankara, Turkey; ^15^Merck, Intercontinental Region, Dubai, UAE; ^16^Department of Neurology, 1st St. Petersburg State Medical University n.a. I.P. Pavlov, St. Petersburg, Russia; ^17^Multiple Sclerosis Center, American University of Beirut Medical Center, Beirut, Lebanon; ^18^Faculty of Medicine, Ain Shams University, Cairo, Egypt; ^19^Department of Neurosciences, King Faisal Specialist Hospital and Research Centre, Riyadh, Saudi Arabia

## Abstract

The burden of multiple sclerosis (MS) in women of childbearing potential is increasing, with peak incidence around the age of 30 years, increasing incidence and prevalence, and growing female : male ratio. Guidelines recommend early use of disease-modifying therapies (DMTs), which are contraindicated or recommended with considerable caution, during pregnancy/breastfeeding. Many physicians are reluctant to prescribe them for a woman who is/is planning to be pregnant. Interferons are not absolutely contraindicated during pregnancy, since interferon-*β* appears to lack serious adverse effects in pregnancy, despite a warning in its labelling concerning risk of spontaneous abortion. Glatiramer acetate, natalizumab, and alemtuzumab also may not induce adverse pregnancy outcomes, although natalizumab may induce haematologic abnormalities in newborns. An accelerated elimination procedure is needed for teriflunomide if pregnancy occurs on treatment or if pregnancy is planned. Current evidence supports the contraindication for fingolimod during pregnancy; data on other DMTs remains limited. Increased relapse rates following withdrawal of some DMTs in pregnancy are concerning and require further research. The postpartum period brings increased risk of disease reactivation that needs to be carefully addressed through effective communication between treating physicians and mothers intending to breastfeed. We address the potential for use of the first- and second-line DMTs in pregnancy and lactation.

## 1. Introduction

Multiple sclerosis (MS) has an average age of onset of about 30 years [1], although studies from various populations indicate earlier presentations [2]. Approximately 5% of patients present before the age of 18 years [3], believed to be associated with a putative environmental factor acting at the age of puberty [4]. The increasing prevalence and incidence of MS, with a 2-3-fold preponderance of cases in women versus men, imply a substantial and growing burden of MS among women of childbearing age [1, 5–7].

MS* per se* does not appear to carry significant risk of an adverse pregnancy outcome, compared with women without MS; indeed, relapse rates are often reduced during pregnancy, although the longer-term clinical disease course is unaffected [7–14]. However, current recommendations for the management of MS propose early intervention with disease-modifying therapy (DMT) for these patients [15], and our understanding of the safety of DMTs in this setting is limited. This review (see the following “*Search Strategy”*) examines the current status of DMT therapy with regard to use in pregnancy and postpartum period.

*Search Strategy*. A PubMed search was conducted for individual DMTs used in the management of MS together with the following search: “multiple sclerosis” AND (“pregnancy OR pregnant OR breastfeeding OR lactation”). Other references came from references lists of identified publications or coauthors' reference collections. Preference was given to discussion of larger, randomised evaluations; however, due to the limited evidence base for some DMTs, smaller studies or case reports are discussed where these provide the only evidence available.

## 2. Managing MS in the Patient Who Is Pregnant or Is Planning a Pregnancy

### 2.1. Advising the Female Patient Planning a Pregnancy

Improvements in counselling of women who are planning or beginning a pregnancy have increased the number of women with MS able to complete a pregnancy successfully [16]. A guideline for the management of MS stresses the need for discussion of how pregnancy might reduce the relapse rate (with a possibility of a transient increase after delivery) [17]. It also highlights the need for a conversation between the patient regarding issues such as the impact of MS on fertility, the child developing MS, treatments (DMTs and others, such as vitamin D), during pregnancy, aspects of the delivery (pain relief and epidural anaesthesia), and postpartum issues such as breastfeeding [17].

The guideline does not specify a need to raise the issue of family planning in a woman of childbearing potential at the time of first prescription of a DMT. Physicians may be divided on the need for this approach: a survey conducted in 2014 of 26 healthcare professionals in the USA working with people with MS revealed that 57% of physicians initiated a discussion on family planning when a young female patient was starting DMT treatment, and a further 28% sometimes do this [18]. Sixty-one percent recommended stopping the DMT when planning pregnancy and conception, and this advice was common to all DMT (79%) and any patient (82%). The opinion of other neurologists was the strongest influence on their advice to pregnant women regarding DMT therapy. For women, broader concerns over the pressure of such a momentous decision at the time of imminent parenthood and attitudes of society towards drug treatment during pregnancy may accompany concerns over maternal and foetal health [19].

### 2.2. Pregnancy and Individual Disease-Modifying Therapies

#### 2.2.1. Evidence Provided in This Section

Key studies are summarised in this section, focusing on larger and more recent studies where available. Information on indications/contraindications is from the European Summary of Product Characteristics for each clinically available drug discussed.  Table 1 provides an overview of which currently available DMTs should or should not be used during pregnancy and lactation, according to European and US labelling.

#### 2.2.2. Interferon-*β*

Interferons are not contraindicated absolutely during pregnancy, and risks and benefits of maintaining treatment during pregnancy (and the potential for relapse from withdrawing treatment from a patient with highly active MS) should be discussed with the patient (Table 1) [20]. The prescribing information for interferon-*β*1_a_ or interferon-*β*1_b_ highlights an increased risk of spontaneous abortion; however, this appears to be based on animal studies, without authoritative support from clinical studies, as described above. Breastfeeding is not supported in Europe during treatment with interferon, as it is not known whether the drug is present in breast milk.

The largest systematic review to date of pregnancies exposed to *β*-interferons found evidence for an association of this treatment with reduced mean birth weight and birth length and preterm birth (<37 weeks); however, there was no increased risk of serious pregnancy complications of spontaneous abortion, Cesarean delivery, or birth weight < 2.5 kg [21]. Data from registry-based cohorts or worldwide drug experience databases suggested no adverse effects of interferon-*β* on pregnancy outcomes, including studies of 425 pregnancies (Rebif® global drug database) [22] and 298 pregnancies (the Avonex® pregnancy registry) [23], up to 423 pregnancies (Betaseron® Pregnancy Registry) [24, 25].

Additional reassuring data are available from registries of 251 births [26] or 78 births [27] in Germany, which demonstrated no effects of interferon-*β* exposure (versus no DMT) during the first trimester on anthropometric parameters at birth, preterm delivery, spontaneous abortion, or the incidence of malformations. A further prospective observational cohort study in Germany (96 pregnancies exposed to interferon-*β*_1a_ or interferon-*β*_1b_ versus 64 pregnancies in MS patients and 1,556 healthy controls) also found no significant impact of interferon on pregnancy outcomes [28]. An observational study from Italy (based on structured telephone interviews) found no evidence for increased risk of spontaneous abortion from 88 pregnancies exposed to interferon-*β* for up to four weeks versus 308 pregnancies not exposed to a DMT; there was a small but significant tendency towards lower birth weight and risk of preterm delivery with interferon, however [29]. Finally, a retrospective study from Italy found that interferon-*β* was associated with a nonsignificant trend towards lower birth weight arising from 38 pregnancies (versus 202 nonexposed pregnancies), but that development up to 18 months was indistinguishable between exposed and nonexposed pregnancies [30].

Two other studies had provided a signal for increased risk of adverse foetal outcomes in patients with MS receiving interferon-*β*. A longitudinal cohort study of only 23 pregnancies found a higher miscarriage rate on treatment (39% versus 5% on no treatment, *p* = 0.03) [31], while a meta-analysis of eight clinical evaluations of interferon-*β*_1a_ (69 pregnancies, including 41 with some exposure to treatment) identified a nonsignificant trend towards spontaneous abortion with the interferon [32].

#### 2.2.3. Glatiramer Acetate

Glatiramer acetate is contraindicated during pregnancy in Europe due to insufficient information on its risk : benefit profile (can be used with caution in the USA) [20]. Breastfeeding while taking this agent is possible, subject to discussion with the patient of risks and benefits to herself and her child [20].

Analysis of 246 pregnancies in people with MS (151 exposed to glatiramer acetate) in the German Multiple Sclerosis and Pregnancy registry revealed no increased risk of spontaneous abortions or other adverse foetal outcomes [33]. Prospective observational data from Italy showed that, among 17 pregnancies exposed to glatiramer acetate, versus 202 unexposed pregnancies, the only significant predictors of spontaneous abortion were higher maternal age and previous pregnancy history; glatiramer acetate was without significant influence on any foetal outcome [34]. Other studies described above (prospective cohort study [28] or registry study [27]) also did not demonstrate clear evidence of harm to the foetus. Similar conclusions relating to the reproductive safety of glatiramer acetate arose from prospective [35] and retrospective [36] case series of MS pregnancies.

Patient preferences are recognised increasingly as an important facet of clinical decision-making. A majority of women planning pregnancy chose to continue glatiramer acetate therapy at least until pregnancy was established and a substantial majority of these women elected to this treatment throughout the pregnancy, without adverse pregnancy outcomes [37, 38].

#### 2.2.4. Natalizumab

Natalizumab causes reproductive toxicity seen in animals, but antibodies cross the placenta only after week 28 of a pregnancy, which marks the initiation of foetal exposure to natalizumab [39]. If natalizumab is continued during pregnancy, it is necessary to monitor the foetus for mild-to-moderate thrombocytopenia and anaemia after delivery [20]. Natalizumab is excreted in breast milk and patients should not breastfeed on treatment, according to EU guidance [20].

Major malformations, and the incidences of low birth weight and premature birth, did not occur at a significantly increased rate in 101 pregnancies exposed to natalizumab during the first trimester, compared with matched controls with MS and a healthy control group [40]. In another study, 28/35 women who accidentally became pregnant while taking natalizumab delivered healthy babies, with one case of hexadactyl, five miscarriages, and one elective termination [41].

A small case series (13 pregnancies in 12 women) suggested that natalizumab is a therapeutic option throughout pregnancy for patients with highly active disease. However, natalizumab was detectable in the blood of the newborns, of whom 10/13 demonstrated haematologic abnormalities [42], apparently confirming earlier case reports of foetal haematologic abnormalities (reduced CXCL12-induced T-cell chemotaxis) during treatment of the mother with this drug [43].

#### 2.2.5. Fingolimod

Observational analysis is available for of 89 pregnancies occurring in clinical evaluations of fingolimod (61 involved first-trimester exposure to fingolimod with known pregnancy outcome) [44]. Pregnancy outcomes included two births with foetal malformations, four terminations due to foetal malformations, and nine spontaneous terminations (plus a further 20 elective terminations). The authors concluded that an absolute contraindication for fingolimod in pregnancy should be in place, given these preliminary data and the known teratogenicity of fingolimod in animals and postmarketing data. Moreover, its labelling specifies maintenance of contraception for up to two months following cessation of fingolimod, due to this agent's persistence in the body for this time [20]. In addition, fingolimod is concentrated in the breast milk of animals, relative to the blood, and patients taking fingolimod should not breastfeed [20].

#### 2.2.6. Teriflunomide

A retrospective analysis of the teriflunomide global pharmacovigilance database showed that 83 pregnancies in women, and 22 pregnancies in partners of men receiving this treatment, led to healthy newborns without reproductive safety concerns [45]. Although preliminary, these data are of interest as teriflunomide has been shown to induce reproductive toxicity in animals and is currently contraindicated during pregnancy [20]. Moreover, contraception is required before/after treatment, as long as the plasma drug level remains above 0.2 mg/L; an accelerated elimination procedure is needed if pregnancy occurs on treatment; patients should not breastfeed while receiving this treatment [20].

#### 2.2.7. Alemtuzumab

Alemtuzumab has demonstrated reproductive toxicity in animals; it is not absolutely contraindicated during pregnancy but should only be given at this time “only if the potential benefit justifies the potential risk to the foetus” (EU and US label) [20]. The drug is excreted in breast milk in animals, but it is unknown whether this occurs in humans; patients should not breastfeed on treatment or for four months after cessation of therapy [20].

A pooled analysis of three Phase 2 evaluations (plus a posttrial extension phase) of alemtuzumab in patients with relapsing-remitting MS (RRMS) has been reported in abstract form [46]. Women who became pregnant were ineligible for further treatment but were followed up for safety (including pregnancy) outcomes. At the time of analysis, the pregnancy outcomes were known, or the pregnancy was still in progress, for 183/193 women who had received alemtuzumab. Two-thirds (66%) of pregnancies resulted in live births free of birth defects. The rate of spontaneous abortions (22%) or stillbirths (1%) was described as comparable to rates seen in untreated MS patients or the general population (22–26%). These data are reassuring, but more study is required. Given the half-life of alemtuzumab, women are advised not to get pregnant for four months after the course of alemtuzumab, and if pregnancy occurred after the first course, the second course of alemtuzumab should be postponed till delivery if breastfeeding is not anticipated.

#### 2.2.8. Mitoxantrone

Information is limited for other DMTs. Mitoxantrone should not be given during pregnancy. Its European labelling recommends maintenance of contraception for at least six months and avoidance of breastfeeding for 28 days after cessation of therapy, while the US label recommends pregnancy testing before each dose [20]. Isolated case reports have described one infant each with (Pierre Robin sequence) or without a significant birth defect in mothers treated with mitoxantrone [47, 48].

#### 2.2.9. Dimethyl Fumarate

Data from clinical trials involving dimethyl fumarate (39 exposed pregnancies with known outcome) did not demonstrate a clear signal for foetal abnormalities (26 live births, three spontaneous abortions, and 10 elective terminations). Similar data were presented for post-marketing observation from 28 exposed pregnancies of known outcome: here, there were only 10 live births and 13 spontaneous abortions. Nevertheless, the authors did not consider there to be “increased risk of foetal abnormalities or adverse pregnancy outcomes associated with gestational exposure” to dimethyl fumarate [49]. This agent is not recommended during pregnancy, though use in pregnancy or breastfeeding is possible subject to risk : benefit considerations [20].

#### 2.2.10. Is It Safe to Withdraw a Disease-Modifying Therapy during Pregnancy?

There are concerns over potential risks of stopping a beneficial DMT during pregnancy. A recent (2015) systematic review found that, due to a potentially high susceptibility of studies to bias and small sample sizes, there is insufficient evidence to quantify either the risk of DMT therapy to the foetus or the risk arising from a temporary suspension of DMT therapy (a “drug holiday”) to the mother [50]. However, this analysis focused on disability and mortality outcomes in patients with RRMS, rather than relapse rates, as well as long-term outcomes in patients with secondary progressive MS. Recent case reports have highlighted concerns over MS recurrence after withdrawal of natalizumab, including a case report suggestive of a rebound in disease activity with new pseudotumoral lesions [51, 52], a series of four pregnant women with increased disability and MRI activity on stopping treatment [53, 54], and a further case involving a young woman with increased disease activity after discontinuing a clinical trial of natalizumab to plan a pregnancy [55, 56]. A similar case was reported with fingolimod [51, 52]. These observations are consistent with the appearance of clinically significant rebound of disease activity, with an average of nine new gadolinium-enhancing lesions and 9 T2 lesions, in 5/46 patients who stopped fingolimod treatment [53, 54].

### 2.3. After the Pregnancy

The health statuses of mother and offspring are key considerations regarding the resumption of DMT therapy following childbirth. Breastfeeding does not appear to be associated with increased risk of postpartum MS relapses and may provide a modest degree of protection from relapses in the mother [9, 55–57] and possibly some protection from MS in adulthood for the child [58]. Accordingly, breastfeeding is highly recommended for a mother with MS, where possible. Given the large numbers of women of childbearing potential with MS, as described above, more research is clearly needed from the pharmaceutical sponsors of these medications to define whether, and in what amounts, they are present in human breast milk (see Table 1).

Anti-inflammatory therapy with corticosteroids is a therapeutic option for the management of MS after delivery. High-dose prednisolone may be given at this time, but breastfeeding should be not undertaken within four hours of administration in order to allow drug concentrations in breast milk to decline sufficiently [59].

## 3. Conclusions and Authors' Recommendations

Further studies are needed to determine the safety and potential risks associated with preconception and exposure to newer DMTs* in utero* in patients with MS. Discontinuation of DMTs before conception remains the general recommendation except in highly active disease, according to individual circumstances (Table 1). Alternatively, a patient receiving a first-line oral DMT with limited safety data (e.g., teriflunomide or dimethyl fumarate) may be switched to a DMT with more safety data available relating to pregnancy, for example, an interferon or glatiramer acetate. Such a switch will depend on the circumstances of the patient, including acknowledgment of their preferences. The views of the authors on the current state of this fast-moving field of MS research, with pragmatic recommendations, based on the clinical evidence base discussed above, are summarised in Additional Points.

For the future, it will be important to collect new data on the use of DMTs in pregnancy. Pregnancy registries, cited above, have provided important information on the safety of DMTs in this setting, with data on a substantial number of pregnancies available particularly for the interferons. The US Food and Drug Administration provided guidelines on the establishment of pregnancy registries in 2002, and such registries are underway for the newer DMTs. It will be important to encourage participation in these registries, to improve data collection on the safety of DMTs in pregnant women with multiple sclerosis, and to deepen the evidence base for therapeutic intervention in this condition.

## Figures and Tables

**Table 1:** Overview of labelling recommendations relating to pregnancy or breastfeeding for disease-modifying therapies for multiple sclerosis.

DMT	Europe (EU)	USA
Interferon^a,b^	No absolute contraindication, discuss risks and benefits with the patient. Discontinue either therapy or breastfeeding.	Pregnancy category C: consider risks and benefits in pregnancy. Apply caution when prescribed for a nursing mother.

Glatiramer acetate^c^	Contraindicated in pregnancy. Consider risks and benefits during breastfeeding.	Pregnancy category B: use “only when clearly needed”. Use with caution during breastfeeding.

Teriflunomide^d^	Contraindicated during pregnancy. Patients must not breastfeed on treatment.	Pregnancy category X: contraindicated. Patients must not breastfeed on treatment.

Dimethyl fumarate^e^	Not recommended in pregnancy. Consider risks and benefits for use during breastfeeding.	Pregnancy category C: consider risks and benefits in pregnancy. Use with caution during breastfeeding.

Natalizumab^f^	Consider discontinuation if pregnancy occurs. Patients should not breastfeed on treatment.	Pregnancy category C: consider risks and benefits in pregnancy. Risks during breastfeeding unknown, no recommendation provided.

Alemtuzumab^g^	Not recommended in pregnancy. Patients should not breastfeed on treatment or for 4 months afterwards.	Pregnancy category C: consider risks and benefits in pregnancy. Contraindicated during breastfeeding: discontinue either breastfeeding or treatment.

Fingolimod^h^	Contraindicated during pregnancy. Contraindicated during breastfeeding.	Pregnancy category C: consider risks and benefits in pregnancy. Contraindicated during breastfeeding: discontinue either breastfeeding or treatment.

Mitoxantrone^i^	Avoid pregnancy during and for at least 6 months after treatment. Do not breastfeed during treatment or for 28 days afterwards.	Pregnancy category D: test for pregnancy before each dose; discuss risks to the foetus if pregnancy occurs. Contraindicated during breastfeeding: discontinue either breastfeeding or treatment.

From European Summaries of Product Characteristics and US full prescribing information for ^a^Rebif (interferon-*β*_1a_), ^b^Betaferon®/Betaseron (interferon-*β*_1b_), ^c^Copaxone®, ^d^Aubagio®, ^e^Tecfidera®, ^f^Tysabri®, ^g^Lemtrada®, ^h^Gilenya, and ^i^Novantrone [20]. Selected information only shown: consult full Prescribing Information before prescribing for a patient.

Pregnancy categories (A, B, C, D, and X, see https://chemm.nlm.nih.gov/pregnancycategories.htm for definitions) in US regulatory documentation are being gradually phased out but are included here as they currently appear in the Prescribing Information for these treatments.

## Additional Points

Summary of authors' recommendations is included as follows.

*Before Pregnancy*. Always discuss potential risks versus benefits of a treatment plan. Include discussion of possible miscarriage and teratogenicity that can happen even in normal pregnancy.

*During Pregnancy*. Evidence-based data supporting the use of DMTs in pregnancy remain limited. The evidence base for use in pregnancy is stronger for interferon-*β* versus other DMTs. Limited available evidence indicates a low risk of foetal harm for glatiramer acetate, dimethyl fumarate, natalizumab, and alemtuzumab (glatiramer acetate is contraindicated during pregnancy in some countries; check local labelling). With regard to individual DMTs during pregnancy, (i) for interferon, glatiramer acetate, or natalizumab, women may continue the DMTs until the pregnancy is confirmed; (ii) for teriflunomide, an elimination process with a follow-up drug level is recommended prior to conception; (iii) for dimethyl fumarate and fingolimod, a 2-month washout is recommended prior to conception; (iv) for alemtuzumab, following labelling requirements (avoid pregnancy for 4 months after a course of treatment) involves avoidance of pregnancy for 16 months from initiation of treatment, as the recommended course involves two treatment cycles 12 months apart.

*After the Pregnancy*. Allow a mother without relapses during pregnancy, and with no active disease on MRI conducted 2–4 weeks after delivery, to breastfeed for 6 months. Women who do not plan to breastfeed may resume their DMT immediately. Interferons, teriflunomide, natalizumab, alemtuzumab, fingolimod, and mitoxantrone are contraindicated during breastfeeding. Uncertainty remains as to whether glatiramer acetate, or dimethyl fumarate, is safe for women who choose to breastfeed.

See earlier sections for supporting evidence and references.
